# Novel immune drug combination induces tumour microenvironment remodelling and reduces the dosage of anti-PD-1 antibody

**DOI:** 10.1038/s41598-025-87344-6

**Published:** 2025-03-15

**Authors:** Takahiro Ozasa, Masao Nakajima, Ryouichi Tsunedomi, Shunsuke Goto, Keishi Adachi, Hidenori Takahashi, Koji Tamada, Hiroaki Nagano

**Affiliations:** 1 Department of Gastroenterological, Breast and Endocrine Surgery, Yamaguchi University Graduate School of Medicine, 1-1-1 Minami-Kogushi, Ube, Yamaguchi 755-8505 Japan; 2 Research Institute for Cell Design Medical Science, Yamaguchi University, Yamaguchi, Japan; 3 Department of Urology, Graduate School of Medical Sciences, Kyushu University, Fukuoka, Japan; 4 Department of Immunology, Yamaguchi University Graduate School of Medicine, Yamaguchi, Japan

**Keywords:** Immune checkpoint inhibitor, LAG3-Ig, Neoantigen peptide, Polyinosinic-polycytidylic acid, Tumour microenvironment, Cancer immunotherapy, Tumour immunology

## Abstract

Immune checkpoint inhibitors (ICIs) are effective in clinical settings; however, they present immune-related adverse effects and financial burden. Although dose reduction of ICIs may mitigate these limitations, it could compromise therapeutic efficacy. Using two adjuvants (poly(I:C) and LAG-3-Ig) combined with three neoantigen peptides (Comb), we examined whether Comb could enhance the efficacy of reduced dose of αPD-1 monoclonal antibody (RD-αPD-1 mAb), which has limited efficacy. In a murine colorectal cancer model using an MC38 cell line, Comb addition to RD-αPD-1 mAb enhanced treatment efficacy. Analysis of the tumour microenvironment (TME) in mice treated with Comb using flow cytometry and single-cell RNA sequencing revealed decreased macrophages with highly expressing immunosuppressive genes and increased plasmacytoid dendritic cells with highly expressing antigen-presenting genes. A potent infiltration of CD8^+^ tumour-infiltrating lymphocytes (TILs) with an effector profile was only observed in RD-αPD-1 mAb with Comb. Additionally, single-cell T cell receptor repertoire analysis underscored an oligoclonal expansion of CD8^+^ TILs following treatment with RD-αPD-1 mAb with Comb. This novel immune drug combination may be a promising strategy for reducing αPD-1 mAb dosage while preserving antitumour efficacy through modulating the TME.

## Introduction

Immune checkpoint inhibitors (ICIs), including anti-programmed cell death protein 1 (PD-1) and anti-programmed death-ligand 1 (PD-L1) antibodies, have revolutionised the treatment of various cancers owing to their potent therapeutic efficacy^1,2^. These antibodies mitigate T cell inhibition by disrupting the interaction between PD-1 on activated T cells and PD-L1, which is expressed on tumour and various immunosuppressive cells within the tumour microenvironment (TME), such as tumour-associated macrophages (TAMs), regulatory T cells, and myeloid-derived suppressor cells (MDSCs)^2–4^. Anti-PD-1 antibody is effective against various cancers, including melanoma, non-small cell lung cancer, and renal cell carcinoma, and is widely used in clinical practice^5–7^. Among them, pembrolizumab has potent therapeutic efficacy in high microsatellite instability (MSI-H) colorectal cancer (CRC)^8^.

Despite the high therapeutic potential of ICIs against MSI-H CRC, concerns include the occurrence of immune-related adverse events (irAEs) and economic burden^9,10^. In particular, the economic burden associated with ICIs as global expenditure on these therapies is expected to double by 2026, making them less cost-effective for certain carcinomas^11,12^. Addressing ICI dosage could potentially mitigate issues related to irAEs and financial constraints; however, real-world clinical evidence supporting dose reduction while maintaining efficacy is scarce^13^. Preclinical models demonstrate enhanced therapeutic efficacy when ICIs are combined with molecularly targeted drugs, vaccines, adjuvants, or radiation therapy^14–17^. However, there is limited research on whether these combinations can compensate for the diminished effectiveness of lower ICI doses.

Previously, we demonstrated that a novel immune adjuvant combination of poly(I:C) and LAG-3-Ig significantly enhances anti-tumour responses in established tumours by promoting the proliferation and preventing exhaustion of tumour antigen-specific T cells in the TME^18^. Poly(I:C) is a double-stranded RNA that engages Toll-like receptor 3 on B cells, macrophages, and dendritic cells (DCs), as well as intracellular proteins, such as MDA5 and RIG-I, thereby activating innate and adaptive immunity^19^. LAG-3-Ig is a soluble recombinant fusion protein combining the extracellular domain of LAG-3 with the Fc region of IgG that competitively attenuates the LAG-3 inhibitory signal in T cells while stimulating DCs and monocytes through MHC class II molecule interactions^20–24^. Our studies suggested the potential of combining these immune adjuvants with HSP70/GPC3 antigen-derived peptides to induce peptide-specific immune reactions against various cancers, including CRC. This combination also increased the numbers of CD8^+^ tumour-infiltrating lymphocytes (TILs)^25,26^.

In this study, we examined whether poly(I:C) and LAG-3-Ig immune adjuvants with neoantigen peptides (hereafter referred to as Comb) can surmount the limited anti-tumour efficacy associated with reduced doses of anti-PD-1 monoclonal antibody (RD-αPD-1 mAb) in mouse CRC tumour models. Additionally, we conducted a combined analysis of single-cell RNA sequencing (scRNA-seq) and single-cell T cell receptor sequencing (scTCR-seq) on the altered tumour-infiltrating immune cells induced by Comb and RD-αPD-1 mAb, offering insights into their synergistic effects.

## Methods

### Mice and cell lines

Female 5–7-weeks-old C57BL/6 mice were purchased from Japan SLC (Shizuoka, Japan). The mice were maintained under specific pathogen-free conditions in our facility, as previously reported^27,28^. A highly immunogenic CRC cell line (MC38), a gift from Prof. T. Ojima, Wakayama Medical University, Japan, was cultured in Dulbecco’s modified Eagle’s medium supplemented with 10% foetal bovine serum (Gemini Bio Products, West Sacramento, CA, USA) and 1% penicillin–streptomycin (Wako, Osaka, Japan) at 37 °C and 5% CO_2_. All animal procedures were carried out in accordance with the guidelines and regulations, and approved by the Institutional Animal Care and Use Committee of Yamaguchi University (Yamaguchi, Japan). This study was carried out in compliance with the ARRIVE guidelines (https://arriveguidelines.org).

### Flow cytometry

Tumour-derived cells were stained for cell surface markers and analysed using a flow cytometer and FlowJo software (Table 1).

**Table 1:** Resource used in this study.

Antibody	Clone	Company
Anti-CD45 BV421	30-F11	eBioscience (San Diego, CA, USA)
Anti-CD3 APC-Cy7	145-2C11	eBioscience
Anti-CD4 AF488	GK1.5	eBioscience
Anti-TIGIT PE	1G9	eBioscience
Anti-TIM-3 PerCP-Cy5.5	B8.2C12	eBioscience
Anti-CD11b PerCP/Cy5.5	M1/70	eBioscience
Anti-CD40L PE	MR1	eBioscience
Anti-Gr-1 PE	RB6-8C5	eBioscience
Anti-MHC class II FITC	M5/114.15.2	eBioscience
Anti-CD11c BV421	HL3	BD Biosciences (Tokyo, Japan)
Anti-CD8α BV510	53–6.7	BD Biosciences
Anti-CD45 APC	30F-11	TONBO (Fremont, CA, USA)
Zombie Yellow viability dye		BioLegend (Tokyo, Japan)
Flow cytometer		Company
EC800		SONY (Tokyo, Japan)
BD LSRFortessa X20 cell analyser		BD Biosciences
Immune adjuvant	dose	Company
Poly(I:C)	50 μg	InvivoGen (San Diego, CA, USA)
LAG3-Ig	1 μg	Adipogen (San Diego, CA, USA)
Peptide	Amino acid sequence	Company
Reps1	AQLANDVVL	SCRUM (Tokyo, Japan)
Adpgk	ASMTNMELM	SCRUM
Dpagt1	SIIVFNLL	SCRUM
Drug	clone	Company
Anti-PD-1 mAb	RMP1-14	BioXcell (Lebanon, NH, USA)
Software	Version	Company
FlowJo software	10.9.0	FlowJo LLC (Ashland, OR, USA)

*PE* phycoerythrin, *APC* allophycocyanin, *FITC* fluorescein isothiocyanate, *AF488* Alexa Fluor 488, *PerCP/Cy5.5* peridinin-chlorophyll-protein complex/cyanine5.5, *APC-Cy7* allophycocyanin-cyanine7.

### Assessing anti-tumour effects of the immune drugs in mice

C57BL/6 mice were inoculated subcutaneously (s.c.) with 1 × 10^6^ MC38 cells in the lateral flank on day 0. On days 5, 12, and 19, the mice were injected s.c. with an immune drug combination (Comb: two adjuvants, Poly(I:C) and LAG-3-Ig, combined with three neoantigen peptides) and/or injected intraperitoneally with αPD-1 mAb at 100 or 200 µg/injection. Details regarding the reagents used are outlined in Table 1. The three peptides used in this study were selected based on their prior identification as neoantigen peptides in the MC38 cell line; Adpgk (ASMTNMELM), Dpagt1 (SIIVFNLL), and Dpagt1 (AQLANDVVL)^29^. Tumour size and mouse survival were assessed twice a week. Tumour growth was periodically measured with digital callipers, and tumour volume calculated as follows: (minor axis of tumour)^2^ × major axis of tumour/2. Mice were euthanized by dislocating their cervical vertebrae after carbon dioxide inhalation as soon as signs of distress were observed or a tumour surpassed 5000 mm^3^.

### Single-cell RNA sequencing

C57BL/6 mice were inoculated with MC38 cells, followed by treatment with RD-αPD-1 mAb monotherapy, Comb monotherapy, RD-αPD-1 mAb with Comb, or without treatment. On day 14, tumours were harvested, minced, and digested with Dri Tissue & Tumor Dissociation Reagent (Cat No. 661563; BD Biosciences, Tokyo, Japan) for 30 min at 37 °C. Mice used for scRNA-seq were prepared by mixing tumours harvested from two mice in each group with the median tumour volume in each group. The digested tissue was passed through a 100-μm cell strainer and stained with Zombie Yellow viability dye and BV421-conjugated anti-CD45 mAb. Live immune cells were isolated via fluorescence-activated cell sorting using an SH800 cell sorter (SONY, Tokyo, Japan). Subsequent preparation and analysis of single-cell libraries and TCR were performed, as previously described^30^. Diversity of the paired TCR αβ repertoire was estimated using the inverse Simpson’s index^31^. The grouping of lymphocyte interactions by paratope hotspots (GLIPH) algorithm was used to identify antigen specificity groups from shared CDR3 amino acid sequence motifs within the TCR β chain^32,33^. CDR3 sequences of TCR β clonotypes were clustered according to their local similarities, which was assumed if specific motifs of more than four amino acids in the CDR3 region were observed^34^. Visualization of the T cell profile in relation to shared TCR β and GLIPH specificities was accomplished using GLIPH version 2^32,33^.

### Statistical analysis

Significant differences between three or more groups or between two groups were evaluated through the Kruskal–Wallis test, followed by Dunn’s multiple comparisons test, using JMP pro 16. For mouse survival, Kaplan–Meier curves were depicted, and the log-rank test used for statistical analysis. A *P*-value < 0.05 was considered statistically significant.

## Results

### Comb potentiates the anti-tumour efficacy of reduced anti-PD-1 mAb dosage against CRC

**Fig. 1:**
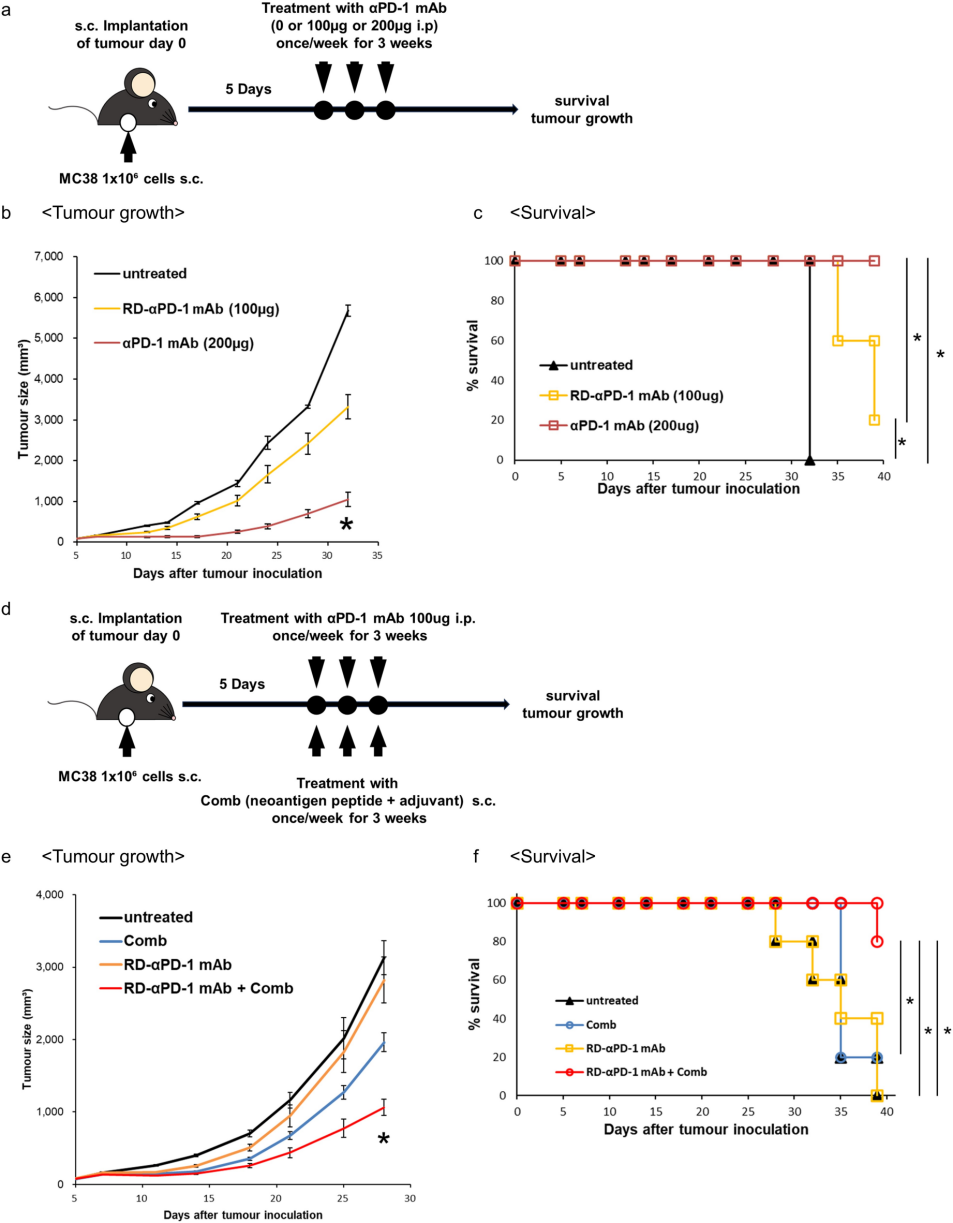
Comb potentiates the therapeutic effect of a reduced dose of anti-PD-1 monoclonal antibody (αPD-1 mAb) against colorectal cancer. (**a**) Schematic of the study design. Mice were inoculated subcutaneously (s.c.) with MC38 and treated with two different doses of αPD-1 mAb (100 or 200 µg/injection) on days 5, 12, and 19. (**b**) The tumour growth of three groups of mice was measured periodically. (**c**) Mouse survival was assessed. (**d**) Schema of the study design using a novel immune drug combination (Comb) and reduced dose of αPD-1 mAb (RD-αPD-1 mAb; 100 µg/injection). Mice were inoculated s.c. with MC38 and treated with RD-αPD-1 mAb monotherapy, Comb monotherapy, and combination therapy on days 5, 12, and 19. (**e**) Tumour growth of four groups of mice was measured periodically (represented in mm^3^). (**f**) Mouse survival was assessed. Representative data from at least two independent experiments are shown. *P < 0.05.

C57BL/6 mice were inoculated s.c. with MC38 cells and treated with anti-PD-1 mAb at 100 and 200 µg (Fig. 1a). A significant anti-tumour effect was observed in mice treated with 200 µg/injection anti-PD-1 mAb at 32 days post-tumour inoculation compared to that in untreated mice (*P* < 0.05) (Fig. 1b). Meanwhile, mice treated with 100 µg/injection anti-PD-1 mAb showed no significant difference in tumour size compared to that in untreated mice (*P* < 0.05) (Fig. 1b). Mice inoculated with MC38 had significantly prolonged survival when treated with 100 and 200 µg/injection anti-PD-1 mAb compared to that of untreated mice. Mice treated with 200 µg/injection anti-PD-1 mAb had significantly prolonged mouse survival compared to those treated with 100 µg/injection (*P* < 0.05) (Fig. 1c). As for irAE, no significant differences in body weight were observed among the three groups (Figure S1a). These findings indicate that a reduced dosage of anti-PD-1 mAb decreased anti-tumour efficacy against CRC.

C57BL/6 mice were inoculated s.c. with MC38 cells and subsequently treated with RD-αPD-1 mAb monotherapy, Comb monotherapy, and combined treatment of both (Fig. 1d). Compared to untreated mice, only RD-αPD-1 mAb with Comb inhibited tumour growth at 28 days after tumour inoculation, and a significantly prolonged mouse survival was observed in mice treated with RD-αPD-1 mAb with Comb compared to that of untreated mice and those treated with each monotherapy (*P* < 0.05) (Figs. 1e, f). No significant differences in body weight were observed among the four groups at 28 days (Figure S1b). These findings indicate that the addition of Comb to RD-αPD-1 mAb substantially improved the therapeutic efficacy against MC38, overcoming the limited anti-tumour efficacy of RD-αPD-1 mAb monotherapy.

### RD-αPD-1 mAb with Comb induces high numbers of CD8^+^ T cells, and Comb modulates myeloid cells in the TME

**Fig. 2:**
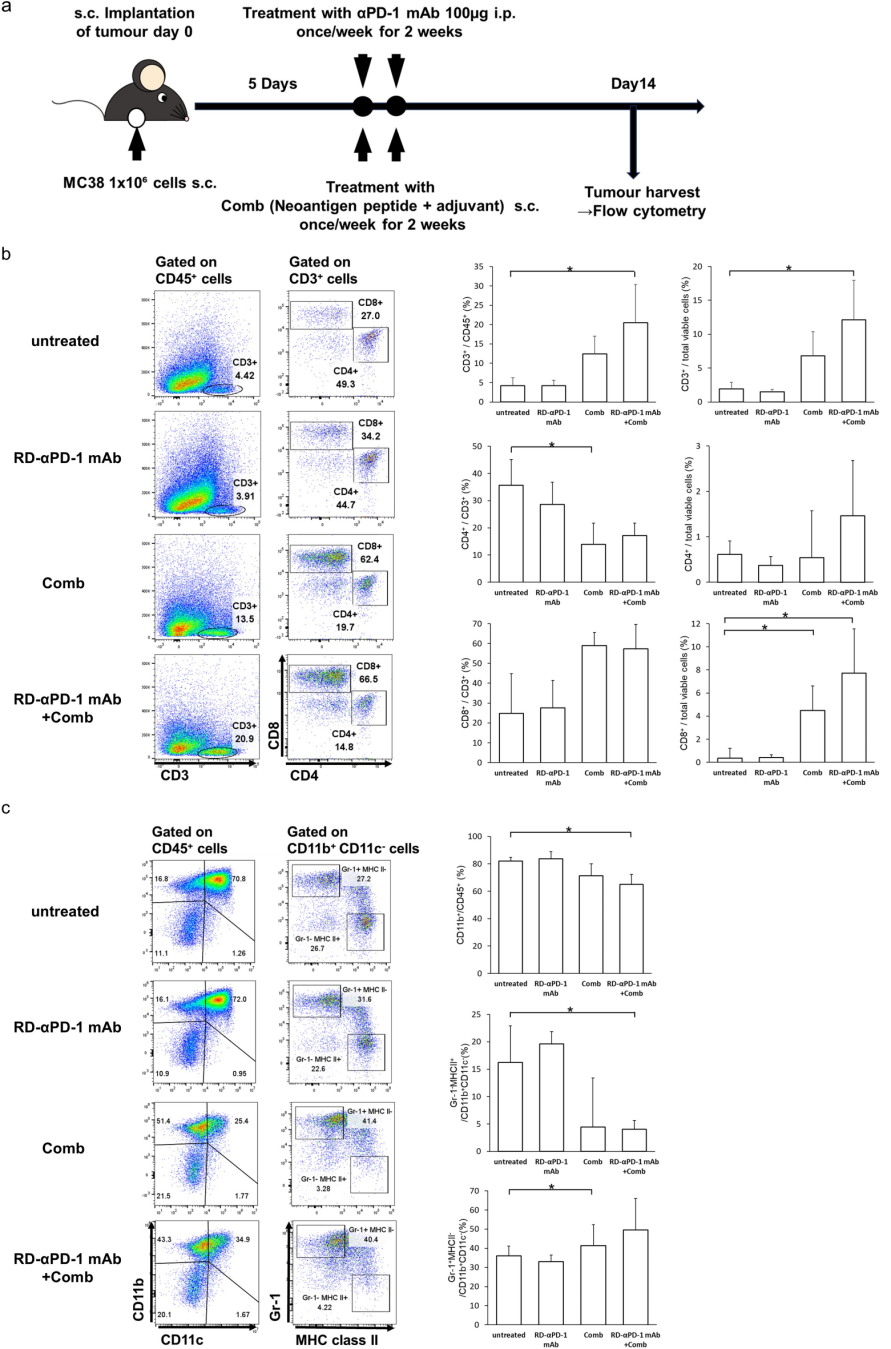
Comb induces a high number of CD8^+^ T cells into tumours and modulates myeloid cells in the tumour microenvironment. (**a**) C57BL/6 mice were inoculated subcutaneously (s.c.) with MC38 on day 0 and treated with reduced dose of anti-PD-1 monoclonal antibody (RD-αPD-1 mAb) monotherapy, the novel immune drug combination (Comb) monotherapy, and RD-αPD-1 mAb with Comb on days 5 and 12. On day 14, immune cells were harvested from tumour tissue and analysed via flow cytometry. (**b**) Proportions of CD3^+^, CD4^+^, and CD8^+^ T cells were assessed, and representative flow cytometric data shown. Data are expressed as the mean ± SD (n = 6). (**c**) Proportions of myeloid cells was assessed, and the representative flow cytometric data shown. Data are expressed as the mean ± SD (n = 6). Representative data from at least two independent experiments are shown. *P < 0.05.

C57BL/6 mice were inoculated s.c. with MC38 cells and treated with RD-αPD-1 mAb monotherapy, Comb monotherapy, and RD-αPD-1 mAb with Comb. CD45^+^ immune cells were harvested from tumour tissue (Fig. 2a). The proportion of CD3^+^ TILs in CD45^+^ cells was significantly higher in mice treated with RD-αPD-1 mAb with Comb compared to that in untreated mice (*P* < 0.05) (Fig. 2b). The proportion of CD8^+^ TILs in total viable cells was significantly higher in mice treated with Comb monotherapy or RD-αPD-1 mAb with Comb compared to that in untreated mice, although the difference was insignificant when the RD-αPD-1 mAb monotherapy group was compared to the untreated mice (*P* < 0.05) (Fig. 2b). Total CD11b^+^ myeloid cells were significantly reduced in the RD-αPD-1 mAb with Comb group compared to that in the untreated group (*P* < 0.05) (Fig. 2c). Moreover, the relative proportion of a Gr-1^−^ MHCII^+^ subset in CD11b^+^ CD11c^−^ myeloid cells was significantly reduced in mice treated with RD-αPD-1 mAb with Comb compared to that in untreated mice (*P* < 0.05). The proportion of a Gr-1^+^ MHCII^−^ subset in CD11b^+^ CD11c^−^ myeloid cells was significantly increased in mice treated with Comb monotherapy compared to that in untreated mice (*P* < 0.05). These findings indicated that the Comb treatment specifically altered the infiltration of T and myeloid cells into the TME. No significant difference was observed in the expression of CD40L, TIGIT, and TIM3 on CD4^+^ and CD8^+^ T cells (Figure S2).

### RD-αPD-1 mAb impacts the exhausted profile of CD8^+^ TILs when used in combination with Comb

**Fig. 3:**
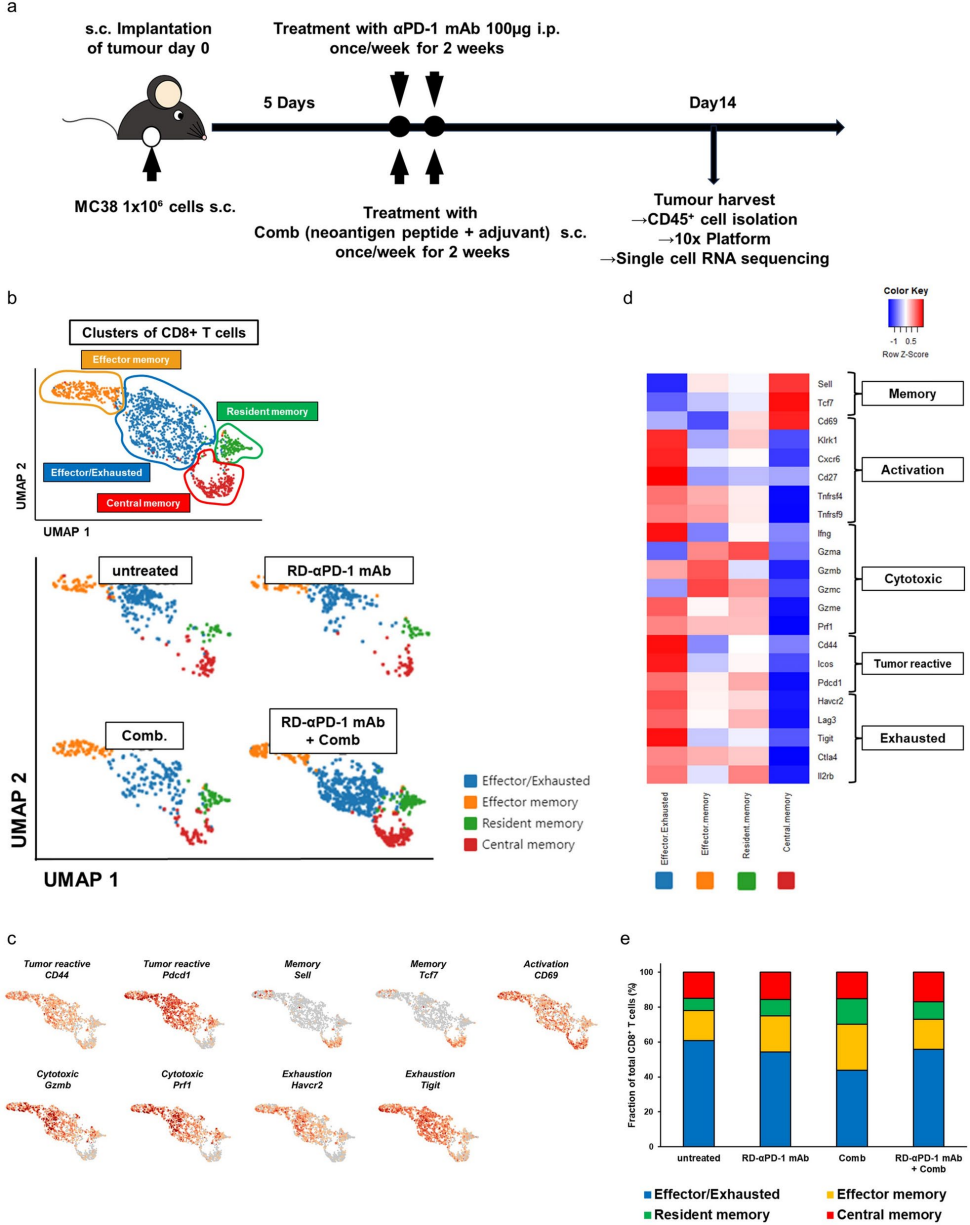

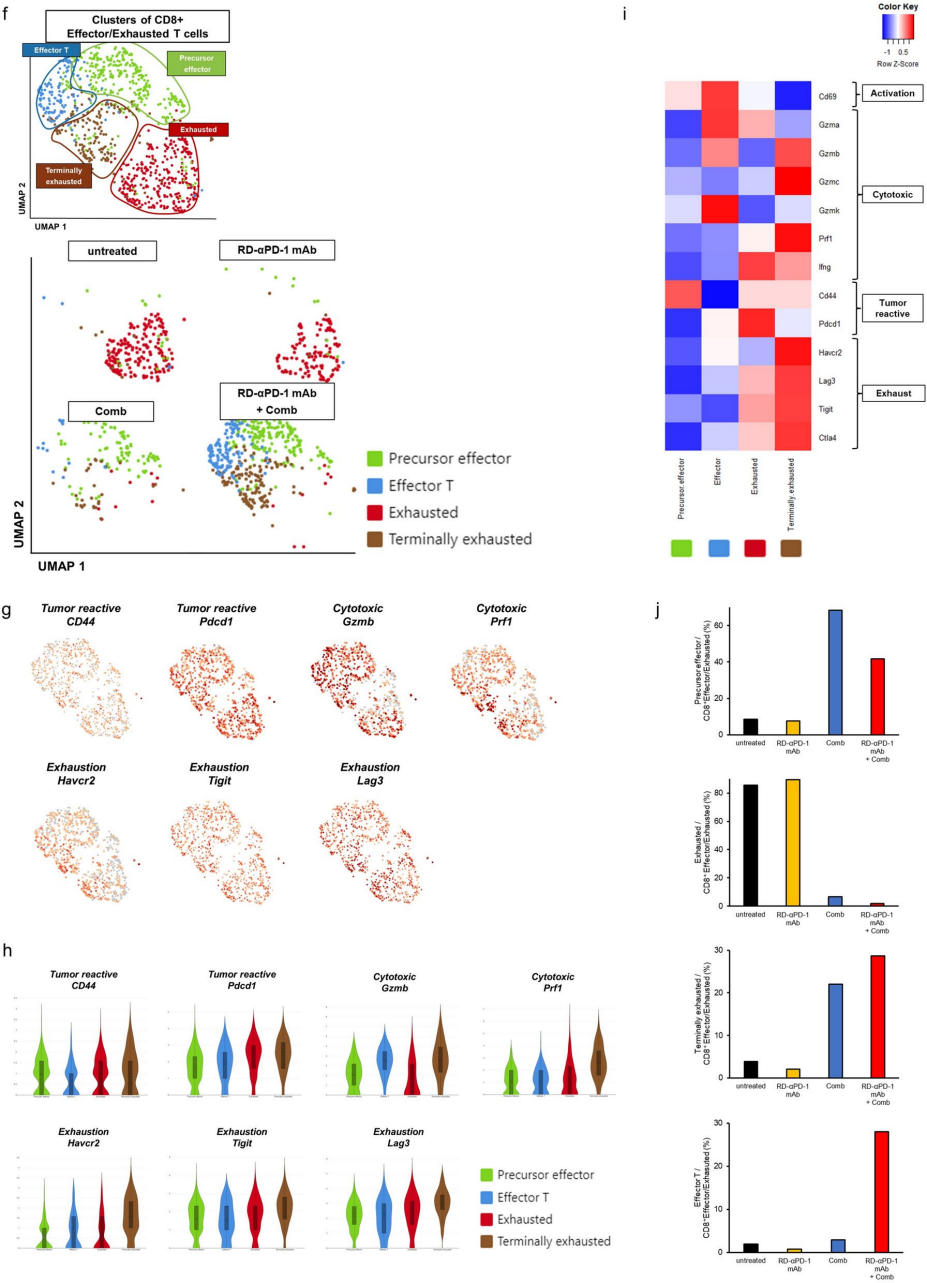
Effects of RD-αPD-1 mAb on the exhausted profile of CD8^+^ tumour-infiltrating lymphocytes when used in combination with Comb. (**a**) Schematic experimental design of single-cell RNA sequencing (scRNA-seq). C57BL/6 mice were inoculated subcutaneously (s.c.) with MC38 on day 0 and treated with reduced dose of anti-PD-1 monoclonal antibody (RD-αPD-1 mAb) monotherapy, the novel immune drug combination (Comb) monotherapy, and RD-αPD-1 mAb with Comb on days 5 and 12. On day 14, immune cells were harvested from tumour tissue for scRNA-seq. (**b**) Uniform manifold approximation and projection (UMAP) plot of CD8^+^ T cells in the tumours of each treatment group. (**c**) Selected genes expressed by profiles of CD8^+^ T cells are overlaid on the UMAP. (**d**) Heatmap displaying the expression of selected genes (row) between cells in each CD8^+^ T cell population (columns). (**e**) Proportion of CD8^+^ T cells of each profile by treatment. (**f**) UMAP plot of CD8^+^ effector/exhausted T cells in the tumours of each treatment group. (**g**) Selected genes expressed by profiles of CD8^+^ effector/exhausted T cells are overlaid on the UMAP. (**h**) Violin plots of selected genes in each CD8^+^ effector/exhausted T cell population. (**i**) Heatmap displaying expression of select genes in each cell population. (**j**) Proportion of individual populations in CD8^+^ effector/exhausted T cells of each treatment group.

For detailed analysis of T and myeloid cells in the TME, scRNA-seq analysis was performed by employing the same mouse model used for flow cytometry analysis (Fig. 3a). Figure S3 shows the flowchart of the uniform manifold and approximation projection (UMAP) plots (Figure S3). CD8^+^ T cells were clustered into four distinct clusters according to the expression of canonical markers for each cell type:^35,36^ effector/exhausted T cells (the antigen expression-related genes, *CD44* and *pdcd1*, along with several exhaustion [*Harcv2*, *Lag3*, *Tigit*, *Ctla4*, and *Il2rb*] and cytotoxic-related genes [*Ifng*, *Gzmb*, *Gzme*, and *Prf1*]), effector memory T cells (*Sell* and cytotoxic-related genes: *Gzma*, *Gzmb*, *Gzmc*, and *Prf1*), resident memory T cells (*CD69* and cytotoxic-related genes: *Gzma*, *Gzmc*, *Gzme*, and *Prf1*), and central memory T cells (*Sell and Tcf7,* without activation and exhaustion-related genes) (Fig. 3b–e). Considering the distinct distribution of effector/exhausted T cells between groups observed in the UMAP analysis, we re-clustered these clusters and identified four subgroups within the CD8^+^ effector/exhausted T cells: precursor effector group (*CD69*, with low expression of cytotoxic and exhaustion-related genes), effector group (cytotoxic-related genes: *Gzma*, *Gzmb*, and *Gzmk*, with low expression of exhaustion-related genes), exhausted group (cytotoxic-related genes: *Gzma* and *Ifng*; and exhaustion-related genes: *Lag3*, *Tigit*, and *Ctla4*, excluding *Havcr2*), and terminally exhausted group (cytotoxic-related genes: *Gzmb*, *Gzmc*, *Prf1*, and *Ifng*; and exhaustion-related genes, including *Havcr2*) (Fig. 3f–i)^37^. Almost all CD8^+^ T cells in mice untreated or treated with RD-αPD-1 mAb monotherapy exhibited an exhausted group. Conversely, mice treated with Comb monotherapy and RD-αPD-1 mAb with Comb exhibited a higher proportion of precursor and terminally exhausted T cells, whereas most effector T cells belonged to mice treated with RD-αPD-1 mAb with Comb (Fig. 3j). Collectively, these results indicate that combining RD-αPD-1 mAb with Comb markedly transformed the exhaustion landscape of CD8^+^ TILs.

### Comb remodels the TME

**Fig. 4:**
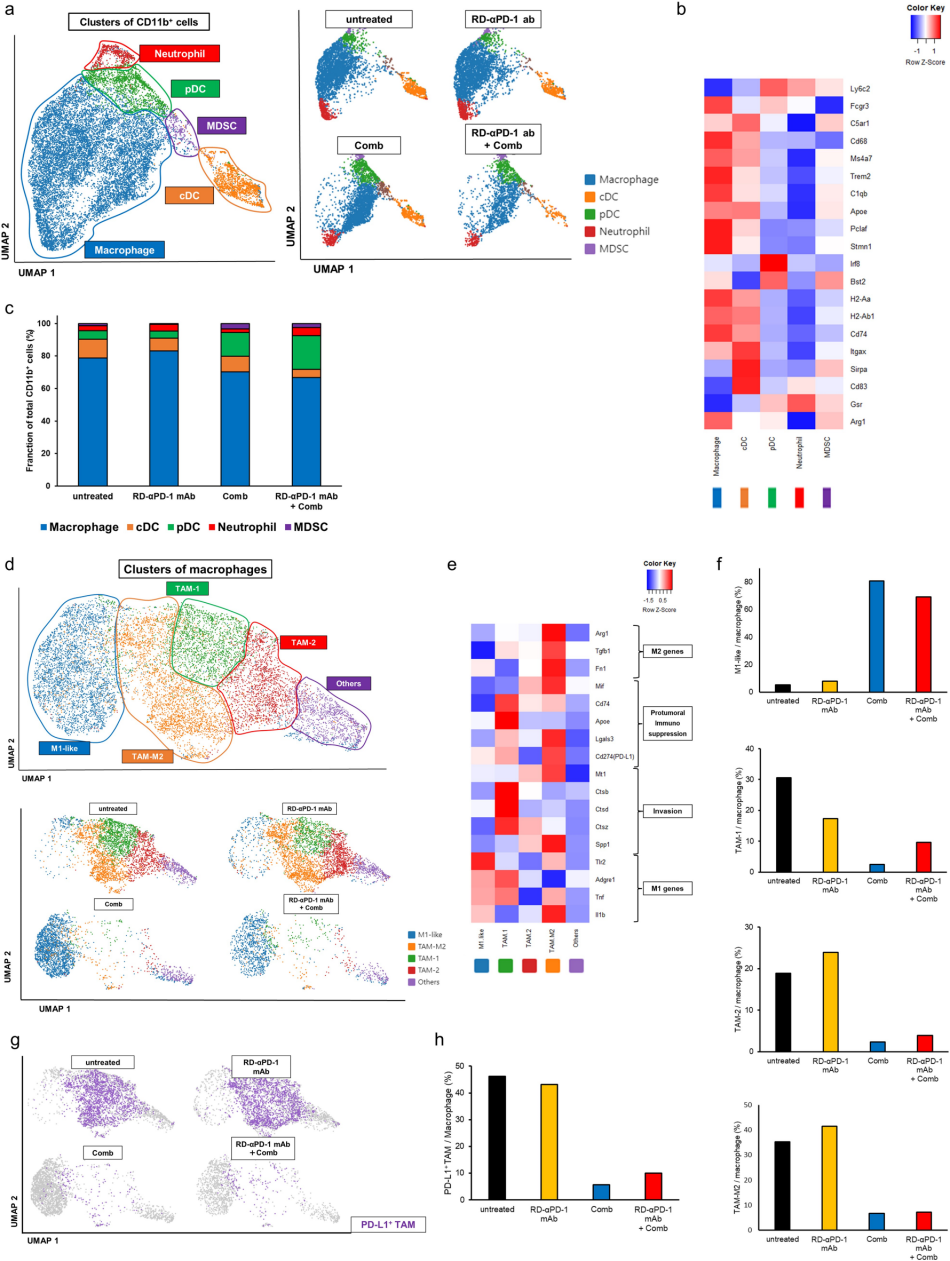

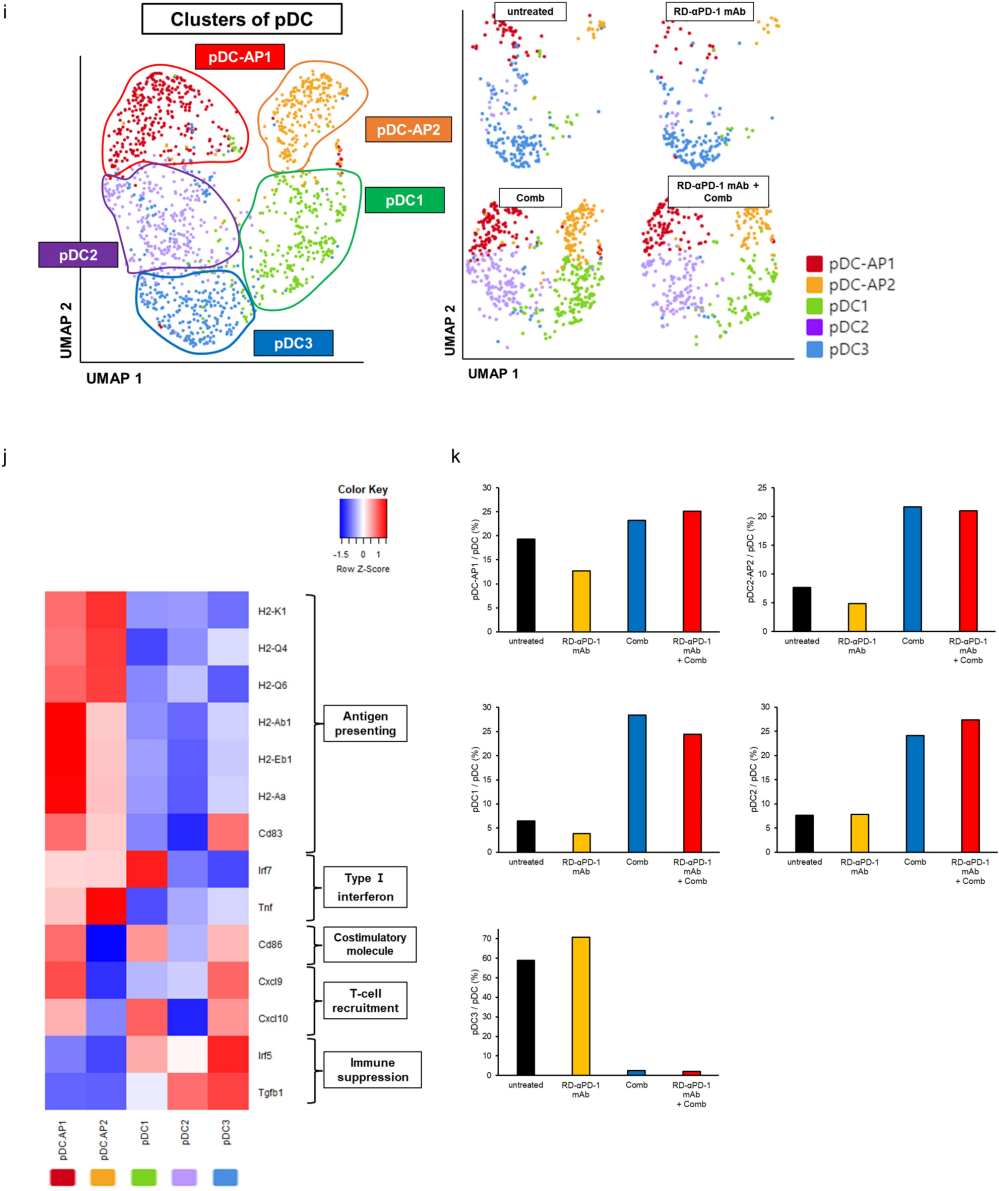
Comb remodels the tumour microenvironment. (**a**) Uniform manifold approximation and projection (UMAP) plots of *itgam*^+^ cells. (**b**) Heatmap of canonical markers identifying specific myeloid subsets. (**c**) Proportion of macrophages, cDCs, pDCs, neutrophils, and MDSCs in each treatment. (**d**) UMAP plots of macrophages. (**e**) Heatmap displaying expression of select genes in five cell populations of macrophages. (**f**) Proportion of M1-like, TAM1, TAM2, and TAM-M2 macrophages in each treatment group. (**g**) UMAP plots of PD-L1^+^ TAM in TAM clusters. (**h**) Proportion of PD-L1^+^ TAM in each treatment group. (**i**) UMAP plots of pDCs. (**j**) Heatmap displaying the expression of selected genes in each cell population. (**k**) Proportion of pDC-AP1, pDC-AP2, pDC1, pDC2, and pDC3 by treatment group. cDC, conventional dendritic cell; MDSC, myeloid-derived immunosuppressive cell; pDC, plasmacytoid dendritic cell; TAM, tumour-associated macrophage.

Re-clustering myeloid cells (itgam^+^) based on gene expression using an unsupervised inference analysis identified 11 clusters of myeloid cells through UMAP dimension reduction (Figure S4a). For simplicity, we organized these clusters into five meta-clusters based on their hierarchical relationships (Figure S4b). Based on the expression of canonical markers, five meta-clusters were observed: macrophages (*CD68*; MHC class II-related genes: *H2-Aa* and *H2-Ab1*; *Trem2*, *C1qb*, and *Apoe*), conventional DCs (cDC; *itgax* and MHC class II-related genes: *Sirpa* and *CD83*), plasmacytoid DCs (pDC; *Ly6c*, *Bst2*, and *Irf8*), neutrophils (*Gsr*, low expression of MHC class II-related genes, and *Arg1*), and MDSCs (*Gsr*, *Arg1*, and low expression of MHC class II-related genes) (Fig. 4a, b)^38^. In Comb monotherapy and RD-αPD-1 mAb with Comb, the proportion of macrophages decreased, whereas that of pDCs increased (Fig. 4c).

Upon further analysis of macrophages through re-clustering, seven clusters were identified and visualized via UMAP dimension reduction (Figure S4c), which were organized into five meta-clusters following their hierarchical ordering based on gene expression (Figure S4d). Five distinct clusters were defined by the expression of canonical markers, including M1-like macrophages, three TAM subpopulations, and others (Fig. 4d, e)^39^. The three TAM subpopulations included TAM-M2 (*Arg1*, *Tgfb1*, and *Fn1*), TAM-1 (*CD74*, *Apoe*, and *Ctsz*), and TAM-2 (*Ctsz* and *Spp1*). M1-like macrophages had high expression of M1-related genes (*Tlr2*, *Adgre1*, *Tnf*, and *Il1b*) and low expression of M2-related and immunosuppressive genes. Almost all macrophages in mice treated with Comb were M1-like macrophages, whereas TAM-M2, TAM-1, and TAM-2 were observed in mice untreated or treated with RD-αPD-1 mAb monotherapy (Fig. 4f). Hence, Comb may remodel the TME by shifting macrophage polarization from M2-like to M1-like states. Additionally, the proportion of PD-L1^+^ TAM was lower in mice treated with Comb and RD-αPD-1 mAb with Comb compared to those untreated or treated with RD-αPD-1 mAb monotherapy (Fig. 4g, h).

In the analysis of pDCs, further re-clustering identified five distinct DC clusters based on the expression of canonical markers (Fig. 4i, j). These included two pDC subtypes with antigen-presenting capacity (pDC-AP, characterized by MHC class I and class II-related genes: *H2-K1*, *H2-Q4*, *H2-Q6, H2-Ab1*, *H2-Eb1*, *H2-Aa*, *CD83*, *IRF7*, and *Tnf*) and three pDC subtypes without antigen-presenting capacity^40^. Among the pDC-AP subtypes, pDC-AP1 was distinguished by high expression of costimulatory molecule (*CD86*) and T cell recruitment chemokine-related genes (*Cxcl9* and *Cxcl10*). Conversely, among the pDCs without antigen-presenting capacity, pDC3 exhibited high expression of immune suppression-related genes (*IRF5* and *Tgfb1)* and low expression of Type I interferon-related genes (*IRF7* and *Tnf*). Notably, most pDC-AP cells were observed in mice treated with Comb and RD-αPD-1 mAb with Comb, whereas nearly all pDC3 cells were found in mice either untreated or treated with RD-αPD-1 mAb monotherapy (Fig. 4k).

### RD-αPD-1 mAb with Comb induces oligoclonal expansion of CD8^+^ TILs exhibiting various memory, activation, and exhaustion profiles

**Fig. 5:**
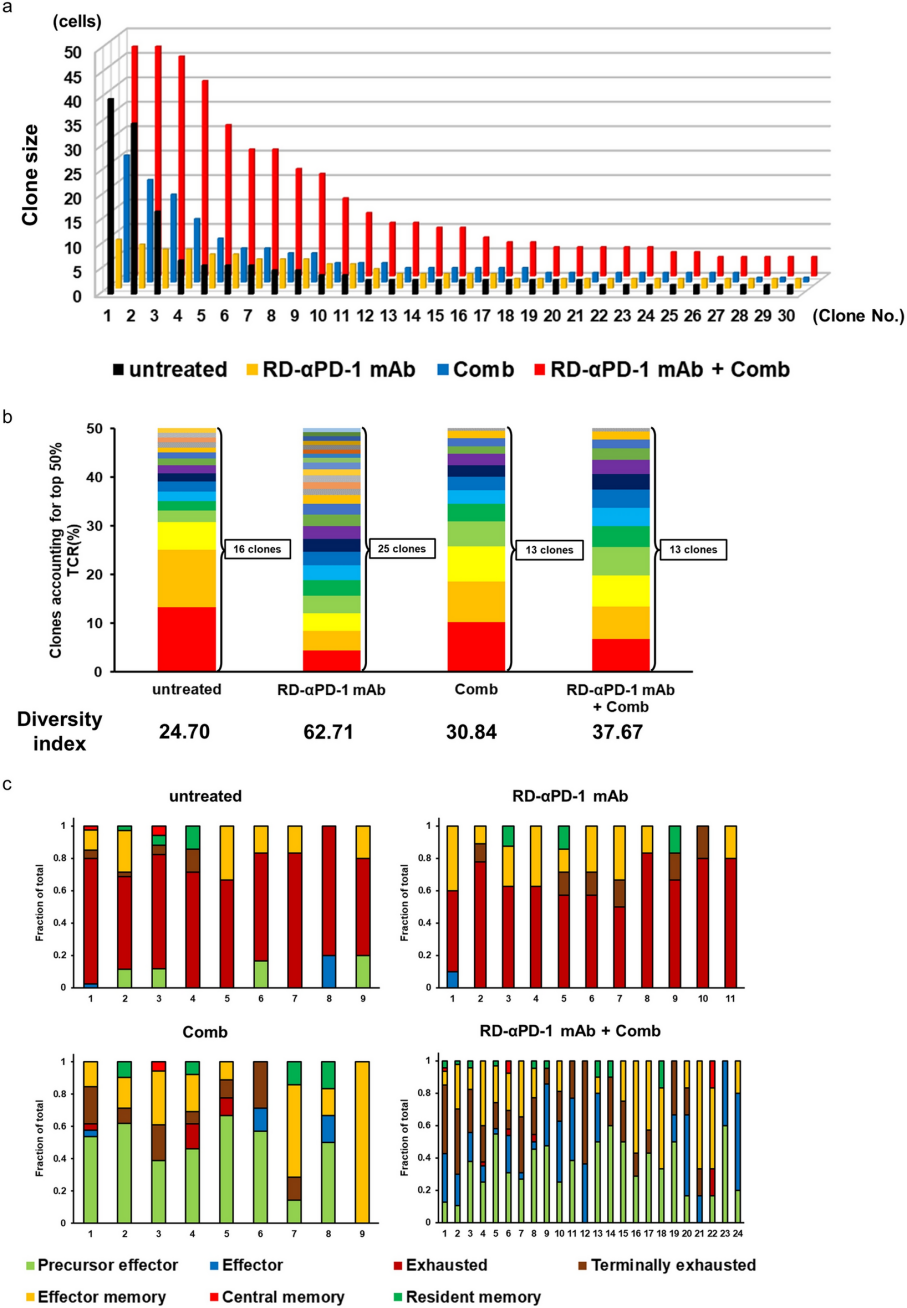
RD-αPD-1 mAb with Comb induces oligoclonal expansion of CD8^+^ TILs with various profiles. (**a**) Bar plot of clonally expanded CD8^+^ T cells of the top 30 most abundant T cell receptor (TCR) clones in each treatment group. (**b**) Number of clones accounting for the top 50% TCR of CD8^+^ T cells, and the diversity of TCRs was calculated using Simpson’s index. (**c**) Relative proportion of each profile of single cells belonging to the same TCR clone larger than five cells for each treatment group.

We generated droplet-based 5′-scRNA-seq and -scTCR-seq libraries to perform a combined analysis of CD8^+^ TIL gene expression and clonal alterations. The RD-αPD-1 mAb with Comb group exhibited an increase in the number of TCR clones that comprise over five cells (24 clones) when compared to that of the untreated (9 clones), RD-αPD-1 mAb monotherapy (11 clones), and Comb monotherapy (9 clones) groups (Fig. 5a and Table 2). We assessed the diversity of the TCR repertoire. The diversity index in the RD-αPD-1 mAb monotherapy-treated mice nearly doubled compared with that in untreated mice (24.70 vs. 62.71) (Fig. 5b). Meanwhile, that of mice treated with Comb monotherapy and RD-αPD-1 mAb with Comb showed a gradual increase compared to that of untreated mice (30.84 and 37.67, respectively).

**Table 2:** Amino acid sequence of the T cell receptor repertoire of CD8 + T cells in each expanded clone.

	Untreated		RD-αPD-1 mAb		Comb		RD-αPD-1 mAb + Comb	
	Clonotype	Clone size	Clonotype	Clone size	Clonotype	Clone size	Clonotype	Clone size
1	CASSRTGGNQDTQYF**	40	CASSQTPGTGGYEQYF	10	CASSQVQGSAETLYF^**§**^	26	CTCSAGPANSDYTF^**†**^	47
2	CASSLELGGREQYF^**‡**^	35	CAWSRQGAHTEVFF	9	CASSRLGGRGDYAEQFF	21	CASSPGQANSDYTF	47
3	CASSRTGGEQDTQYF; CASSRTGGNQDTQYF**	17	CASRRGQEGTLYF	8	CASSRTGGNQDTQYF**	18	CAWSLGGQNTLYF	45
4	CASSQEQGGAETLYF	7	CASSWDWGVNYAEQFF	8	**CASSRLGPSAETLYF**^**¶**^	13	CASSDARAGNTLYF	40
5	CASSRTGGNQDTQYF**	6	CASGKGWGISAETLYF	7	CASSQEGANTEVFF	9	CASSLRENTEVFF	31
6	CASSINWRAETLYF	6	CAWSPGYNSPLYF	7	CASSQDWGTSAETLYF	7	CAWSLGGNSPLYF	26
7	CTCSAGQANSDYTF^**†**^	6	CASSQVQGVGNTLYF^**§**^	6	CGATGQGSGNTLYF	7	CASSDAQWSTLYF	26
8	CAWSPQGAGTGQLYF	5	CASSRRTGVNSDYTF	6	CASSLELGGPEQYF^**‡**^	6	CASSQVQGGQDTQYF^**§**^	22
9	CASSPVGGRQDTQYF	5	CASSLNPGGTYEQYF	6	CASSERLGGRQNTLYF	6	CASSLELGGREQYF^**‡**^	21
10			**CASSRLGPSAETLYF**^**¶**^	5			CASSDGTGSTGQLYF	16
11			CASSIAAQGASGNTLYF	5			CASSQPGQNTEVFF	13
12			CASSQTPGTGGYEQYF	10			CASSWDWGGDTQYF	11
13			CAWSRQGAHTEVFF	9			CASSQALGGDTQYF	11
14							CASSQVQGSAETLYF^**§**^	10
15							CASSLTGGANQAPLF	10
16							CASSTRGREQYF	8
17							CASSLELGGLEQYF; CASSPGGASAETLYF^**‡**^	7
18							CASSLELGGLEQYF^**‡**^	7
19							CAWSRGYNSPLYF	6
20							CASSIRGRWDTQYF	6
21							CTCSAGQANSDYTF^**†**^	6
22							CTCSVTGGMATGQLYF	6
23							CTCSAGRDRAGERLFF	5
24							CASGEPRDFYEQYF	5

Amino acid sequences of the T cell receptor β-chain with a clone size > 5 are shown. Those of the TCR that recognize the same or similar antigens evaluated using GLIPH2 are shown with the same symbol, and each symbol indicates sites where the amino acid sequences are matched by GLIPH2.

RD-αPD-1 mAb, reduced dose of anti-PD-1 monoclonal antibody; Comb, novel immune drug combination; **, CASSRTGGNQDTQYF; ^¶^, CASSRLGPSAETLYF; ^**‡**^, SLELGG*E; ^**†**^, SAG*ANSD; ^**§**^, QVQG.

We further analysed the gene expression profile of CD8^+^ T cells with the same TCR clones that comprised over five cells in each group. Based on the seven clusters used in Fig. 3, the exhausted profile constituted a large proportion of each TCR clone in mice untreated and treated with RD-αPD-1 mAb monotherapy (Fig. 5c). Conversely, CD8^+^ TILs of each TCR clone in the Comb monotherapy group mainly consisted of precursor effector and terminally exhausted or effector memory profiles, whereas those in the RD-αPD-1 mAb with Comb group exhibited a diverse range of profiles, including precursor effector, effector, terminally exhausted, and effector memory profiles. Finally, we identified “TCR specificity groups”— clusters of distinct TCR sequences that likely recognise the same or similar antigens— through shared motifs in the CDR3 sequence, using the GLIPH algorithm on TCR clones (Table 2). Three specific TCR groups among the seven abundant TCR β CDR3 sequences were exclusively observed in mice treated with RD-αPD-1 mAb with Comb.

## Discussion

The optimal dosage of αPD-1 mAb for each patient remains uncertain given the distinctive mechanism of action of ICIs, which differs from traditional chemotherapies. Some irAEs are related to the dose of ICI used; however, reducing ICI doses to mitigate irAEs consequently limits therapeutic efficacy^41,42^. A preclinical model reported decreased anti-tumour efficacy when the ICI dose was reduced from 200 to 100 µg, mainly owing to the insufficient concentration of αPD-1mAb in the TME^13^. In the current study, we confirmed the limited treatment efficacy of RD-αPD-1 mAb against CRC, where the addition of Comb to RD-αPD-1 mAb enhanced the anti-tumour effect without the body weight loss suggestive of severe irAE. Given that Comb costs approximately $30/injection in clinical practice, using it with a RD-αPD-1 mAb is more cost-effective than administering a full dose of αPD-1 mAb^25,26^.

The scRNA-seq data revealed several possible mechanisms underlying the treatment efficacy observed. Comb could alter the polarity of macrophages in the TME. The TME in solid tumours is polarized toward M2 macrophages, which are characterized by the inhibition of T cell activation through cytokine release, exosome secretion, and expression of immune checkpoint ligands^43–47^. Poly(I:C) in combination with other drugs, such as anti-PD-L1 antibodies and ferumoxytol, acts on macrophages in the TME and induces M1 macrophages^48–51^. Additionally, pDCs secrete type I interferon and activate CD8^+^ T cells^40^. In this study, pDCs expressing type I interferon-related genes (*Irf7* and *Tnf*) increased in mice treated with Comb. Considering that Poly(I:C) and LAG-3Ig have an activating effect on pDC through MHC class II-binding, Comb may have synergistically acted on macrophages and pDCs^52,53^. ICIs are less effective owing to suppressive immunity, including M2 macrophages, in the TME^54^. In this study, the high percentage of M2 macrophages in myeloid cells following ICI monotherapy suggests that the reduced ICI dose could not contribute to T cell activation owing to suppressive immunity. Furthermore, PD-L1^+^ TAMs suppress T cells in the TME through the PD-1/PD-L1 axis^46,47^. Herein, PD-L1^+^ TAMs decreased following Comb administration, suggesting that a reduced dose of αPD-1 mAb enhanced the cytotoxic activity of tumour-reactive T cells through TME remodelling induced by Comb.

The anti-tumour effect of ICI depends on the existence of CD8^+^ T cells with less exhaustion^4,55,56^. In this study, precursor effector T cells with low expression of exhaustion-related genes increased in mice treated with Comb owing to the partial improvement of exhaustion by LAG3-Ig^18^. When RD-αPD-1 mAb was added, an increase in effector CD8^+^ T cells with enhanced cytotoxic activity and fewer exhaustion-related genes was observed. These results suggest that RD-αPD-1 mAb could enhance the cytotoxic activity of tumour-reactive, less exhausted T cells induced by Comb through TME remodelling, reducing the required dose of αPD-1 mAb. On the contrary, high expression of *pdcd1* in exhausted CD8^+^ T cells, observed in mice without treatment or those treated with RD-αPD-1 mAb, may contribute to an increase in the amount of αPD-1 mAb required to fully activate these cells.

The successful eradication of tumour cells is associated with the appropriate clonality and various profiles of TILs as essential components of the immune response^34,57,58^. Combined analysis of scRNA-seq and scTCR-seq revealed the possibility that oligoclonal expansions of CD8^+^ T cells with various profiles only occur in mice treated with RD-αPD-1 mAb with Comb. Oligoclonal expansion of the TCR repertoire is correlated with clinical treatment efficacy and patient prognosis^59–61^. Moreover, GLIPH analysis on expanded clones suggested that seven TCRs may recognise three identical antigens in mice treated with RD-αPD-1 mAb with Comb. Among them, two specific TCR groups (shared motifs in the CDR3; QVQG and SLELGG*E) likely recognising the same or similar antigens were also present in the Comb monotherapy group, and one specific TCR group (shared motifs in the CDR3; SAG*ANSD) was also present in the untreated group. This indicates that TILs recognising neoantigen peptides and present before treatment may have been amplified by RD-PD-1 mAb. Meanwhile, the 17 other types of expanded TCRs in the RD-αPD-1 mAb with Comb did not match any of the TCRs from the other treatment groups, suggesting the possibility that they recognise entirely different antigens. Moreover, these clones possess various profiles, including the effector type, suggesting a high likelihood of tumour antigen-specific responses. This may indicate the potential induction of antigen spreading or clonal replacement, where T cells are induced against various tumour antigens by combination of RD-αPD-1 mAb with Comb^62–65^. Additionally, an increase in antigen-presenting pDCs in the combination treatment group suggests that enhanced antigen presentation capability at the tumour site may also be involved in antigen spreading^66,67^. Further in vitro and in vivo experiments are warranted to confirm this hypothesis.

Our study had some limitations. First, the lack of a direct comparison within the same experimental setting to confirm whether the low-dose anti-PD-1 plus comb can fully match the standard-dose efficacy. Although separate data, Fig. 1b, e, suggest comparable antitumour effects, these were not derived from a single controlled experiment and must be interpreted with caution. Second, changes in the TME with Comb identified through scRNA-seq require further analysis to elucidate the mechanisms involved. Third, there are limitations when evaluating irAEs using mouse models, and a detailed evaluation using preclinical models, such as cynomolgus monkeys, is needed. Fourth, because our study focused on a single cell line and its specific neoantigen peptides, it remains an open question whether this combination therapy can be extrapolated to other defined neoantigen peptides or even general tumour-associated peptides. Further studies involving additional cell lines and neoantigen targets will be needed to validate and extend our findings, although ICIs has been used as tumour agnostic treatment.

This study demonstrates that the novel immune drug combination consisting of poly(I:C), LAG-3-Ig, and neoantigen peptides may reduce the required dose of αPD-1 mAb by improving the TME and maintaining antitumour effects.

## Supplementary Information

Supplementary Information.

## Data Availability

The data that support the findings of this study are available from the corresponding　author upon reasonable request.
