# Supplementary material for: Novel immune drug combination induces tumour microenvironment remodelling and reduces the dosage of anti-PD-1 antibody
Source: Sci Rep. 2025 Mar 15;15:8956. doi: 10.1038/s41598-025-87344-6 (PMC11910518; doi:10.1038/s41598-025-87344-6)
Supplement: Supplementary file 1 — Supplementary Information. [file 41598_2025_87344_MOESM1_ESM.docx]

**Supplementary Information**

**Novel Immune Drug Combination Induces Tumour Microenvironment Remodelling and Reduces the Dosage of Anti-PD-1 Antibody**

Takahiro Ozasa, Masao Nakajima, Ryouichi Tsunedomi, Shunsuke Goto, Keishi Adachi, Hidenori Takahashi, Koji Tamada, Hiroaki Nagano

**
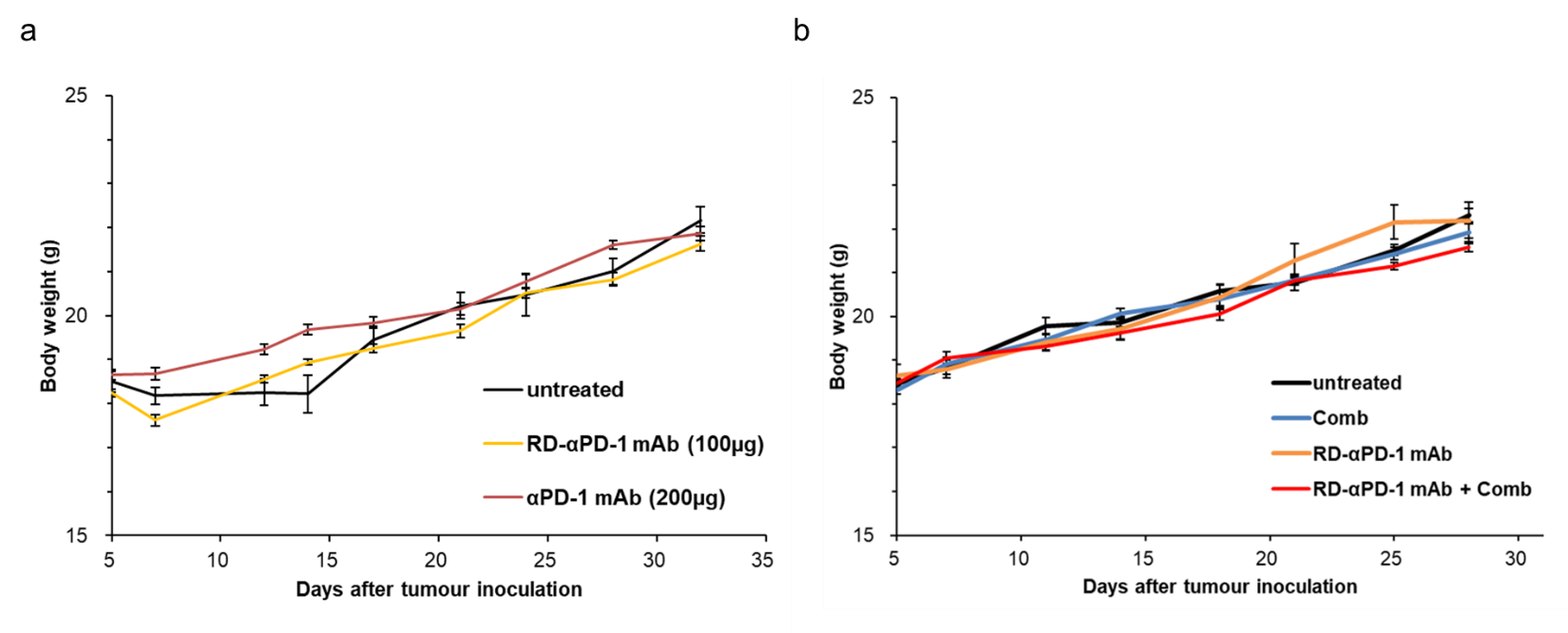
**

**Figure S1. Body-weight monitoring in each experimental group.** (**a**) Mice were inoculated subcutaneously (s.c.) with MC38 and treated with two different doses of αPD-1 monoclonal antibody (mAb) (100 or 200 µg/injection) on days 5, 12, and 19. The body weight of three groups of mice was measured periodically. (**b**) Mice were inoculated s.c. with MC38 and treated with reduced dose of αPD-1 mAb (RD-αPD-1 mAb) monotherapy, the novel immune drug combination (Comb) monotherapy, and combination therapy of RD-αPD-1 mAb with Comb on days 5, 12, and 19. The body weight of four groups of mice was measured periodically.


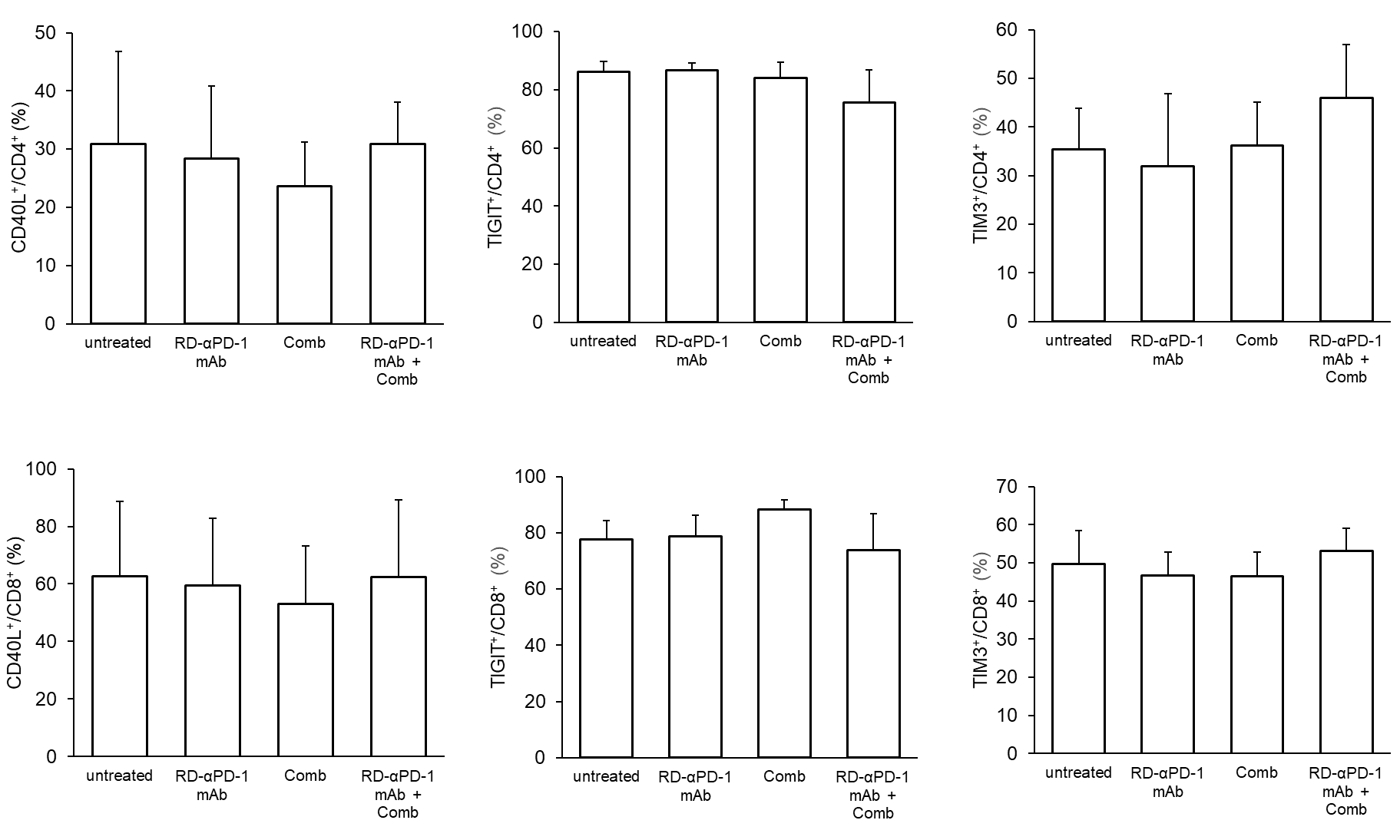


**Figure S2.** **Expression of CD40L, TIGIT, and TIM-3 on CD4^+^ and CD8^+^ T cells of tumour-infiltrating lymphocytes (TILs).** C57BL/6 mice were inoculated subcutaneously with MC38 on day 0 and treated with reduced dose of αPD-1 monoclonal antibody (RD-αPD-1 mAb) monotherapy, the novel immune drug combination (Comb) monotherapy, and combination therapy of RD-αPD-1 mAb with Comb on days 5 and 12. On day 14, tumour-infiltrating immune cells were analysed via flow cytometry. Expression of CD40L, TIGIT, and TIM-3 on CD4^+^ and CD8^+^ T cells were assessed. Data are expressed as the mean ± SD (n = 6). Representative data from at least two independent experiments are shown. *P <0.05.


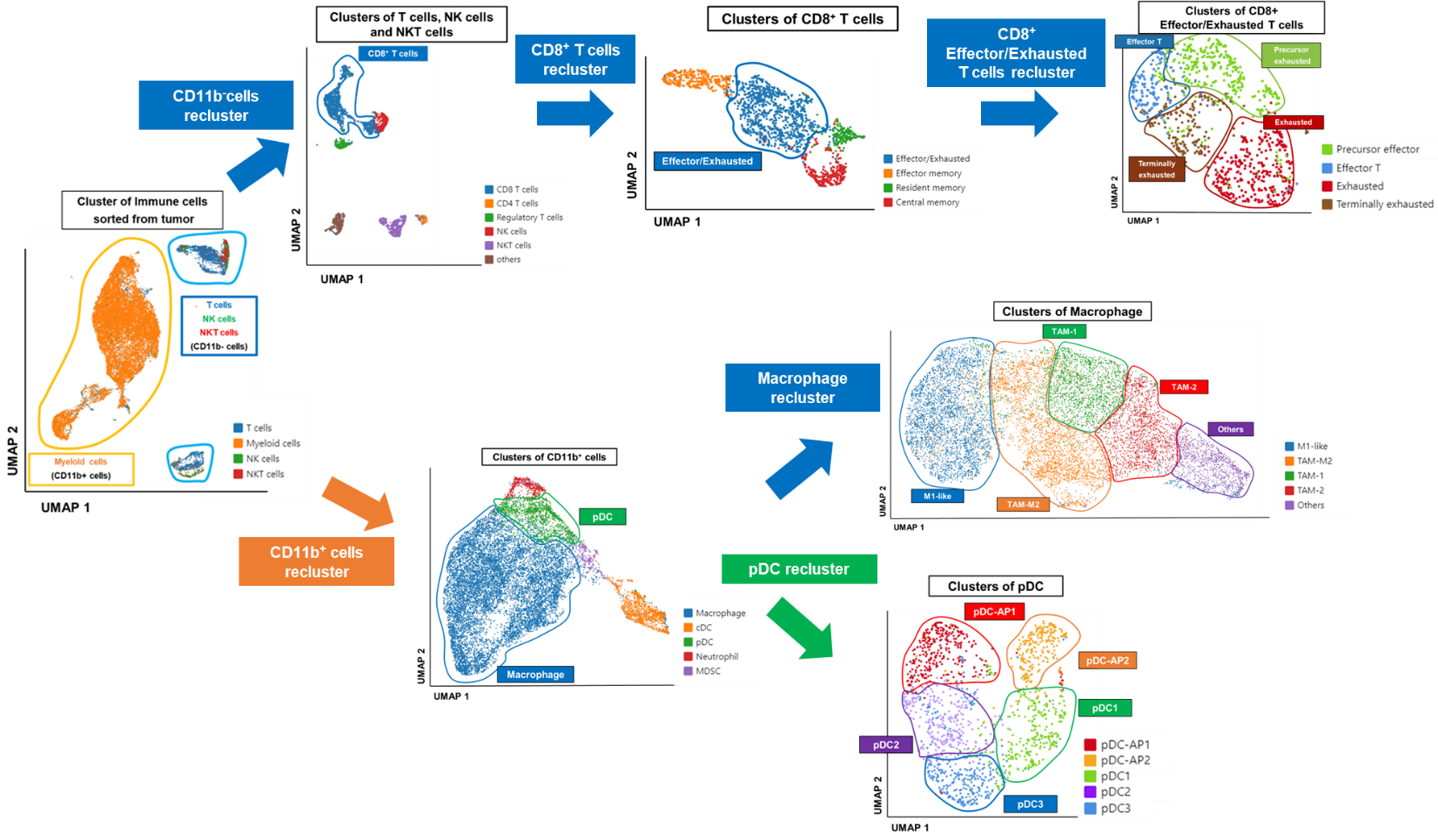


**Figure S3. Overall flow chart of the uniform manifold approximation and projection (UMAP) plots. cDC, conventional dendritic cell; MDSC, myeloid-derived immunosuppressive cell; pDC, plasmacytoid dendritic cell.** Overall flow chart of the UMAP unsupervised clustering process of tumour-infiltrating immune cells according to classical markers. As shown in the flow chart, the immune cells were divided into myelocytes (itgam^+^) and lymphocytes (itgam^−^), which were clustered and classified in detail.

**
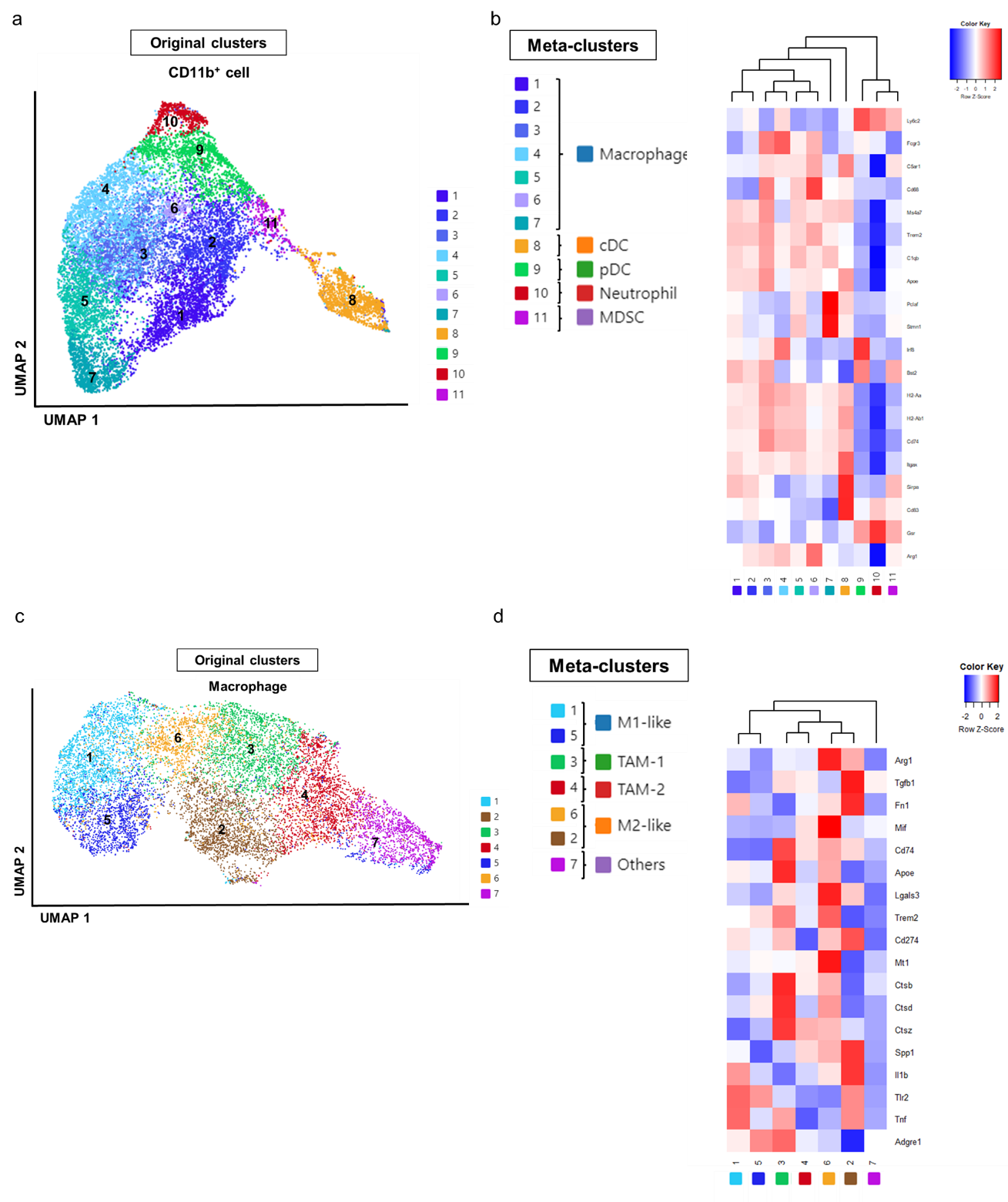
**

**Figure S4. Meta-clusters are based on their hierarchical relationships. cDC, conventional dendritic cell; MDSC, myeloid-derived immunosuppressive cell; pDC, plasmacytoid dendritic cell.** (**a**) Uniform manifold approximation and projection (UMAP) plots show the original 11 clusters of itgam^+^ myeloid cells identified by the Loupe browser after filtering out contaminating lymphocytes. (**b**) Generation of five meta-clusters based on hierarchical ordering. (**c**) UMAP showing the original seven clusters of macrophages identified by the Loupe browser. (**d**) Generation of five meta-clusters based on hierarchical ordering.
